# Changes in leaf chlorophyll content associated with flowering and its role in the diversity of phytophagous insects in a tree species from a semiarid Caatinga

**DOI:** 10.7717/peerj.5059

**Published:** 2018-06-29

**Authors:** Leandro Sousa-Souto, Adriana Bocchiglieri, Douglas de M. Dias, Anthony S. Ferreira, José P. de L. Filho

**Affiliations:** 1 Programa de Pós-graduação em Ecologia e Conservação, Universidade Federal de Sergipe, São Cristóvão, SE, Brazil; 2 Programa de Pós-graduação em Ecologia, Conservação e Manejo da Vida Silvestre, Universidade Federal de Minas Gerais, Belo Horizonte, MG, Brazil; 3 Programa de Pós-graduação em Ecologia, INPA, Manaus, AM, Brazil

**Keywords:** Inflorescence, Chlorophyll content, Phenology, *Poincianella pyramidalis*, Tropical dry forest

## Abstract

Phytophagous insects choose their feeding resources according to their own requirements, but their feeding preferences in the semiarid Caatinga have rarely been studied. Flowering trees leads to a greater diversity of flower visitors and their predators in the host plant, but little is known about why the diversity of phytophagous insects not associated with flowers is also increased. The purpose of this study was to evaluate the diversity of sap-sucking, wood-boring and leaf-chewing insects associated with leaf chlorophyll content in flowering and non-flowering plants of *Poincianella pyramidalis*, an endemic tree of Caatinga. We used a leaf chlorophyll index (LCI) as a surrogate for resource quality, and an entomological umbrella to collect phytophagous insects. We show that trees which bloomed demonstrated higher chlorophyll content, greater abundance and a significant difference in the composition of phytophagous insect species when compared to non-flowering trees (*p* < 0.05). The results suggest that not only the presence of flowers themselves, but also the higher nutritional quality of leaf tissue, can explain the differences in species diversity and abundance of phytophagous insects. Exceptional flowering trees in the Caatinga area studied may thus act as spots of high quality resources, favouring changes in the diversity of insects in this environment.

## Introduction

In the semiarid Brazilian Caatinga, the availability of high-quality resources for insects is concentrated in a short period of time, generally three to four months in the year (the rainy season). This seasonality results in less phytophagous insect diversity, even when compared to other dry forest ecosystems (Leal et al., 2016). It is well recognised that the availability of resources exerts a strong influence on the growth and distribution of herbivore populations (Power, 1992), but quality of resources is crucial to enable persistence and population growth (Kaspari & Yanoviak, 2001). Some characteristics of the host plant affect the quality of its tissues, thus increasing the abundance and species richness of insects (Strong, Lawton & Southwood, 1984; Hunter & Price, 1992; Mendes, Bueno & Carvalho, 2005).

Studies addressing insect-plant interactions with regard to the availability/quality of resources have shown that carbohydrates and nitrogenous compounds are limiting factors that are essential to insects (White, 1978). Nitrogen is usually the most limiting nutrient for plant growth in tropical forests (Sinclair & Horie, 1989), and changes in its availability have a strong effect on the photosynthetic capacity of canopy leaves (Kull, 2002).

In many species, photosynthetic rate is strongly correlated with foliar nitrogen, and the relationship between leaf photosynthetic production and nitrogen content, expressed per unit of leaf area, is generally linear (Sinclair & Horie, 1989; Djumaeva et al., 2012). The relative chlorophyll content (RCC) in plant leaves is therefore a powerful indicator of foliar nitrogen content, and can be determined by direct and indirect methods (Hoel & Solhaug, 1998; Argenta et al., 2001; Percival, Keary & Noviss, 2008; Djumaeva et al., 2012). In general, plants subjected to stress (hydric or nutritional), are more subject to herbivory than those that are not stressed, due to a higher concentration of soluble nitrogen and lower concentration of defence compounds, as secondary metabolites (White, 1984; Cornelissen & Stiling, 2005).

The concentration of soluble nitrogen varies among plant tissues, being higher in structures such as flowers, pollen and leaves, than in the xylem and phloem. Since nitrogen content may indicate plants with higher nutritional quality for herbivores (Eubanks, Styrsky & Denno, 2003), plant structures with high nitrogen levels may attract a greater diversity of insects (Cunha et al., 2006).

Plants in Caatinga generally flower in the rainy season, or exceptionally in the dry season, after sporadic rains, with individuals blooming throughout the year (Amorim, Sampaio & Araújo, 2009; Leite & Machado, 2009). Flowering asynchrony leads to a mosaic distribution of high-quality resources, with some plants acting as oases amid an array of non-flowering plants. Flowering can affect the presence of insects in multiple ways, such as through visual attraction, or as shelter and food for herbivores, predators and parasites (trophic interactions). Nevertheless, asynchronous blooming during the dry season, may be an indicator of a better nutritional status in the host plant, and can attract phytophagous insects that are not directly related to the flower’s presence, such as bark and wood-boring, sap-sucking and leaf-chewing insect assemblages (Augspurger, 1981).

The present study investigates the role of plant nutritional status in the diversity of phytophagous insects in individuals of *Poincianella pyramidalis* (Fabaceae), during the dry season. We tested whether flowering plants harbour a high species richness and abundance of phytophagous insects (which are not directly associated with the presence of flowers) when compared to non-flowering plants, and whether this might be due to their nutritional status (using RCC as surrogates for nitrogen content). Due to their higher levels of chlorophyll, flowering individuals can harbour different species composition, as well as a greater abundance and species richness of phytophagous insects (sap-sucking, bark and wood-boring and leaf-chewing) than non-flowering individuals.

## Material and Methods

### Study species

The *P. pyramidalis* (Fabaceae) tree, popularly known in Brazil as “catingueira” is endemic, and one of the most abundant trees in our study area (Oliveira et al., 2013; Sousa-Souto et al., 2014). The species is found in the states of Alagoas, Bahia, Ceará, Paraíba, Pernambuco, Piauí, Rio Grande do Norte and Sergipe (Maia, 2004), and flowers irregularly in the Caatinga (Leite & Machado, 2009). Individuals of *P. pyramidalis* have a cluster-type inflorescence and the flowers are dioecious and zygomorphic (Miranda & Gimenes, 2013), with a high concentration of nectar, mainly in the days following the opening of the flower bud (Leite & Machado, 2009). This species has been used extensively in the semiarid region of Brazil for the production of coal, wood, and forage for cattle and sheep (leaves).

### Study area

**Figure 1:**
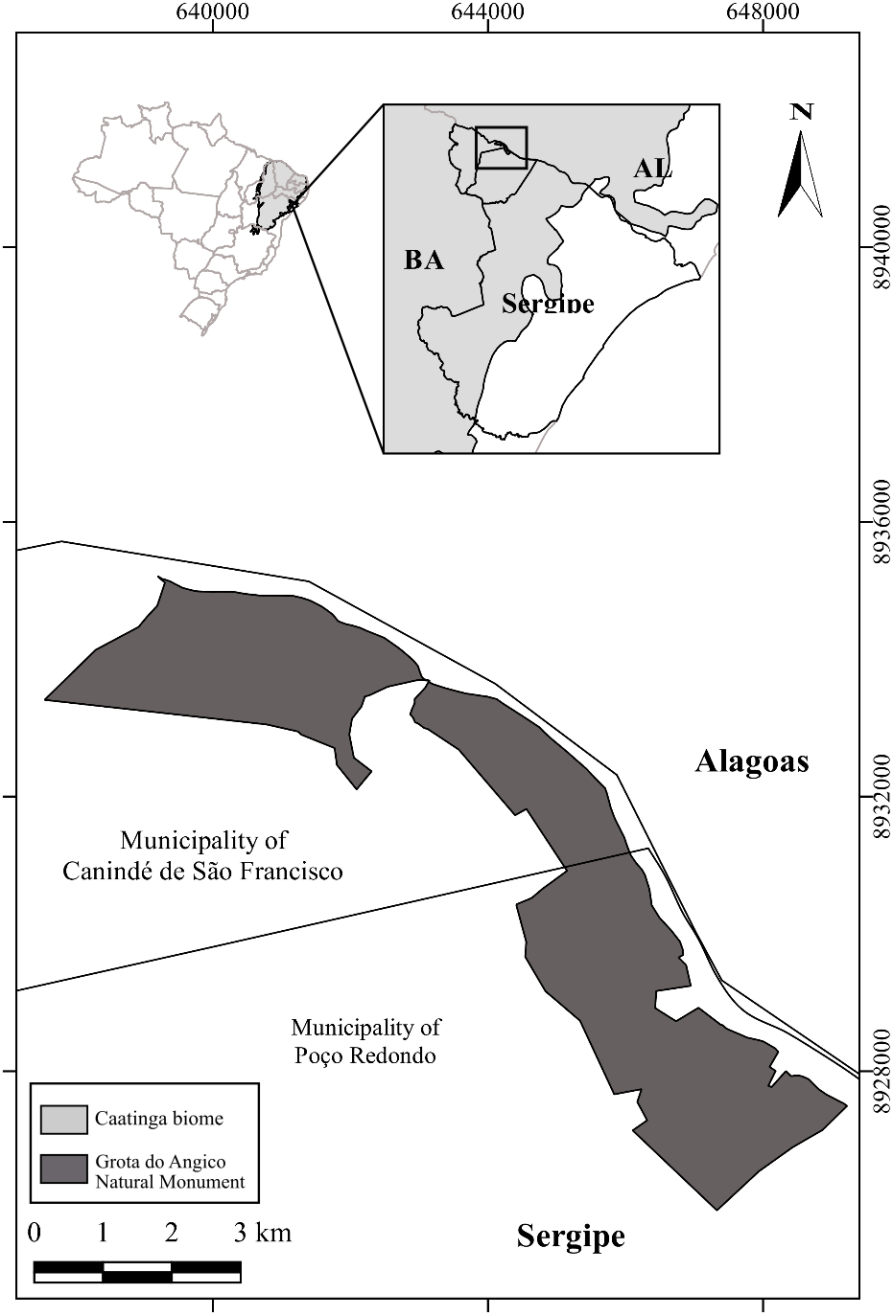
The study area. Monumento Natural Grota do Angico—MNGA (dark gray), and the occurrence of the Caatinga Biome (light gray). The MNGA covers approximately 2,183 ha and is located between the municipalities of Poço Redondo and Canindé de São Francisco, northeastern Brazil. Map created in *QGis 2.14 Essen* (http://www.fsf.org/).

The study was conducted in the “Monumento Natural Grota do Angico (MNGA)” conservation unit, located in the municipalities of Poço Redondo and Canindé de São Francisco, in the state of Sergipe (9°39′S; 37°40′W) (Fig. 1), and covering about 2,183 ha (field permit #2011.04.1008/00123-021/SEMARH/SE). The climate is classified as tropical semi-arid (BShw - Köppen), with low and irregular average annual rainfall (about 500 mm/year). The dry season generally lasts some seven to eight months a year, from September to April (Silva, Prata & Mello, 2013). The vegetation is semiarid and deciduous xerophytic, with a tree layer containing 174 species belonging to 51 families, ranging from 7 m to 15 m (Silva, Prata & Mello, 2013). The MNGA has several fragments of secondary forest in diverse stages of succession with approximately 15 years of forest regeneration, surrounded by pastures (Ribeiro et al., 2013; Silva et al., 2013).

### Sampling design

We sampled 60 adult individuals of *P. pyramidalis* in an area of approximately 2 ha. We selected 30 trees in bloom and 30 other individuals that were not flowering, with similar diameter, canopy area, height and leaf density. Because *P. pyramidalis* is a deciduous species, the peak of leaf production occurs at the beginning of the rainy season (Parente et al., 2012), and thus, leaf age (senescence) between plants with and without flowers, at the time of sampling, was considered the same. Sampling was performed in November 2012 (dry season) and due to the low density of flowering individuals, was systematic. For each flowering tree sampled, we located the nearest tree without flowers with a circumference at breast height (CBH) > 5 cm for additional sampling, with a minimum distance of 10 m between individuals within pairs.

### Assessment of relative chlorophyll content (RCC)

Samples were taken over three consecutive days, with 10 flowering and 10 non-flowering trees sampled per day. Each collection was made within a maximum period of two hours (between 9 am and 11 am) to avoid large variations in temperature and humidity throughout the day, which could substantially alter the leaf chlorophyll values between the samples.

We sampled the first leaflet in the terminal third of three branches exposed to solar radiation for each *P. pyramidalis* selected. The branches selected for the evaluation of chlorophyll were located at the base of the canopy at a height between 1.5 and 2 m. We evaluated only mature leaves, with no sign of senescence, signs of predation or attack by microorganisms. We took three readings per plant (three leaves per tree) with a chlorophyll meter mark ClorofiLOG^®^ model CFL 1030, which provides measurements of chlorophyll a, b and total (*a + b*) contents, expressed in Leaf Chlorophyll Index (LCI) units (Barbieri Junior et al., 2012). The procedure was repeated on the 60 sampled trees (with and without flowers), obtaining a total of 180 LCI measurements. The average value of the chlorophyll was then calculated for each tree.

### Arboreal-insect sampling

About 15 min after the three chlorophyll samples were taken in the branches we started collecting insects. We used the beating technique with an entomological umbrella to quantify the number of species, composition and abundance of phytophagous insects. The sampling session consisted of 10 hits in each of the previously-sampled branches, totalling 30 hits per tree (see Sousa-Souto et al., 2014 for details). After beating, all the insects present in the inner area of the umbrella were collected, packed in plastic bags with ethyl acetate, and identified to the lowest possible taxonomic level using entomological keys (Triplehorn & Johnson, 2005; Rafael et al., 2012). Only adult phytophagous insects not directly related to the flowers were considered for this study, and insects were grouped into three feeding guilds, based on their mouthparts morphology (bark and wood borers, leaf-chewing and sap-sucking insects), following Rafael et al. (2012). Only families with those three predominant feeding habits were included in the analyses. We consider these guilds only, due to their different resource use and probable sensitivity to changes in the chlorophyll content in plant tissues.

Due to the scarcity of data for the insect taxonomy of the Caatinga, we separated insects by family and identified morphospecies within each family. Insects identified as ”bark and wood-boring”, and “leaf-chewing” belonged to the Coleoptera order. The sap-sucking insects consisted of adults of the suborders Auchenorrhyncha, Sternorrhyncha and Heteroptera (Rafael et al., 2012).

To compare the LCI in trees with and without flowers, we used the Wilcoxon test (*α* < 0.05), as data was not normally distributed (Crawley, 2013), with the assumption that flowering plants had a better nutritional status (high quality of food resource). We used the Wilcoxon test (*α* < 0.05) to compare the richness and abundance of phytophagous insects between trees with and without flowers. We also used generalised linear models (GLMs), corrected with quasi-Poisson error structure, to test the relationship between species richness and/or abundance of insects and chlorophyll content, regardless of plant status (with or without flowers), where the richness and abundance of insects were variable responses and the LCI was the explanatory variable. Minimal models were adjusted by excluding non-significant variables and verifying effects on deviance (Crawley, 2013).

To test for possible differences in functional groups composition between trees with and without flowers, we used non-metric multidimensional scaling (NMDS) (Nascimento et al., 2014). The ordination was based on the Bray Curtis index (abundance data). We used a similarity analysis (ANOSIM; Clarke, 1993) to compare the difference between the two groups. Differences in R values obtained were used to determine the dissimilarity of the patterns for both flowering and non-flowering plants. All analyses were performed in the statistical platform R 3.1 (R Core Team, 2017).

## Results

**Figure 2:**
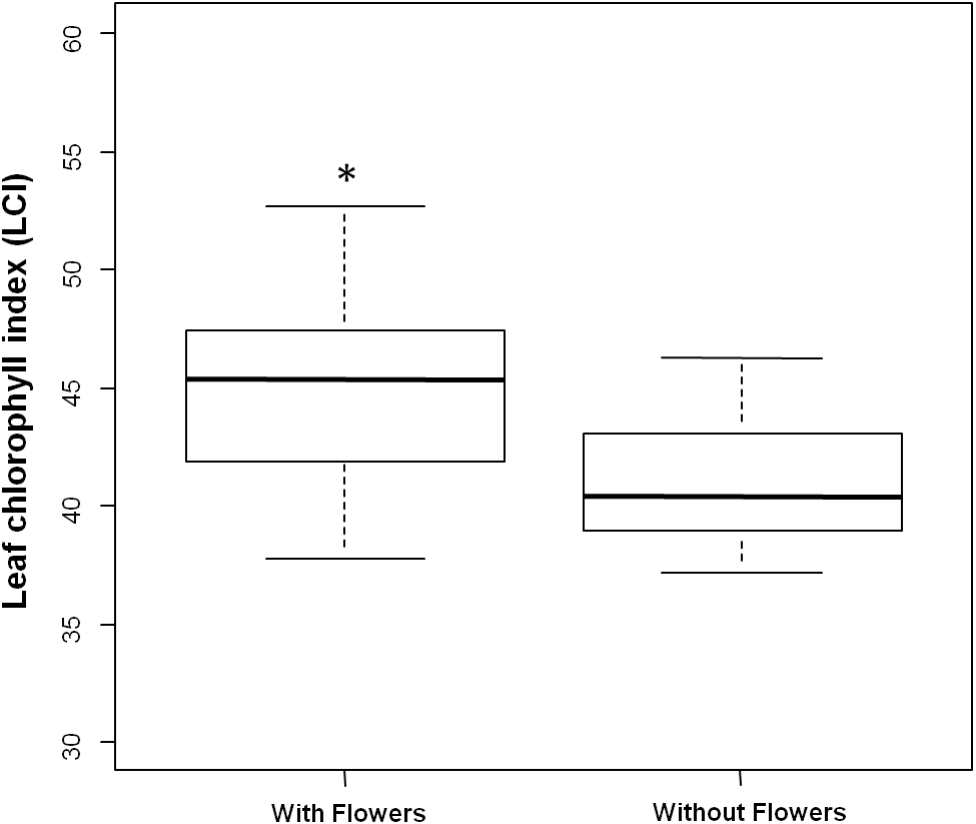
Leaf chlorophyll index (LCI) between the two groups of trees (flowering and non-flowering). Difference in the leaf-chlorophyll index (LCI) of trees with and without flowers. Median (black vertical line) and interquartile range (boxes), as well as the maximum and minimum values are shown. Asterisks indicate significant difference between the two groups of trees (*p* < 0.05).

**Figure 3:**
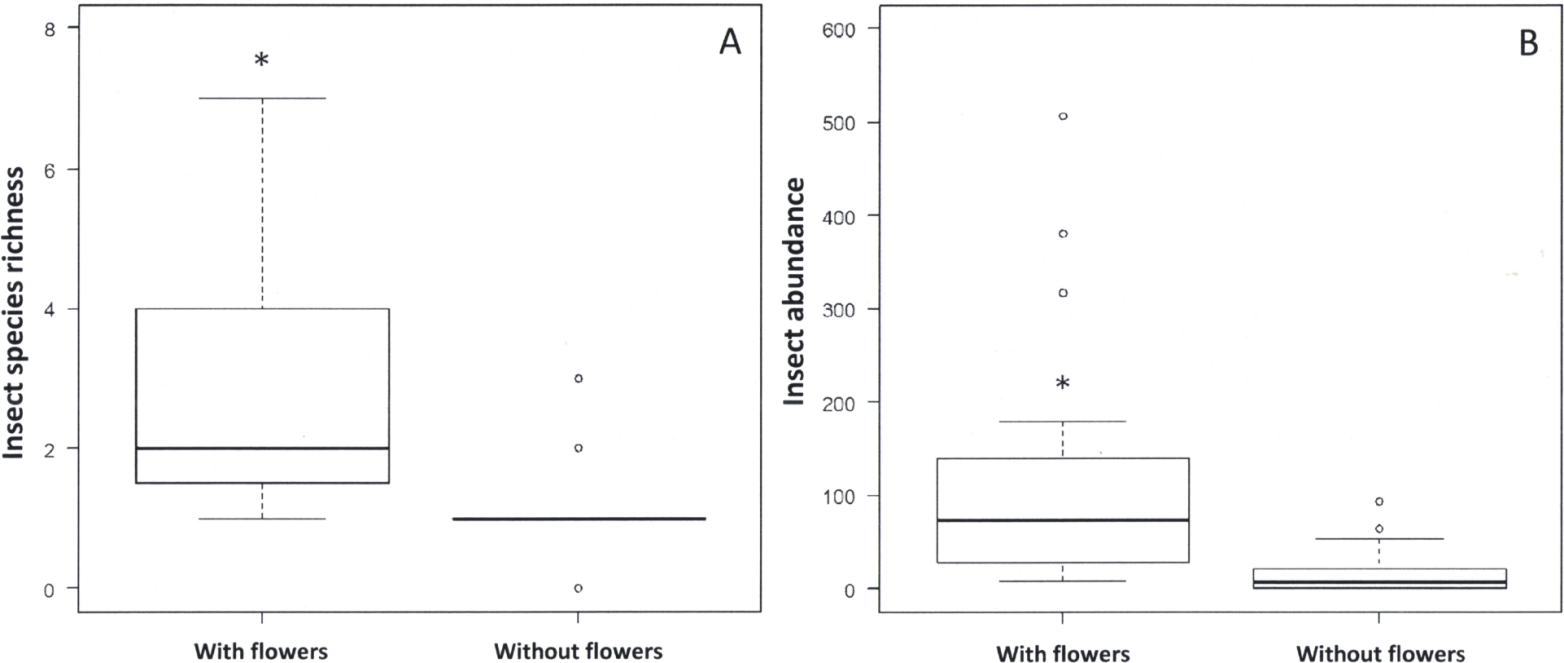
Richness and abundance of insects among flowering and non-flowering plants. Median (black vertical line), interquartile range (boxes) and maximum and minimum values of insect species richness (A) and insect abundance (B) on plants with and without flowers in the Monumento Natural Grota do Angico. Asterisks indicate significant difference between the two groups of trees (*p* < 0.05).

Flowering trees had higher chlorophyll content, expressed as LCI (*W* = 551; *p* < 0.01), than trees without flowers (Fig. 2). Species richness (*W* = 578.5; *p* < 0.01) and abundance (*W* = 625.5, *p* < 0.01) of phytophagous insects were also higher in blooming trees than in trees without flowers (Fig. 3).

We sampled a total of 3,734 arboreal insects, distributed in five orders (Blattodea, Coleoptera, Diptera, Hemiptera and Hymenoptera), comprising 21 families and 50 morphospecies. Considering only the three functional groups analysed, 3,480 individuals (27 morphospecies) were sampled, of which 2,957 were captured in blooming trees and 472 in non-flowering ones (Table S1). The individuals of these functional groups were distributed in two orders: Coleoptera (with five families and 20 morphospecies) and Hemiptera (four families and seven morphospecies). The greatest richness was observed in wood-boring insects, *Sibinia* sp. and *Sibinia hirritus* (Coleoptera: Curculionidae) were the most abundant morphospecies, representing 88% and 10%, respectively, of the insects sampled. In total we sampled 13 bark and wood-boring morphospecies, seven sap-sucking and seven leaf-chewing morphospecies. There were 23 morphospecies in flowering trees (19 were exclusive) and seven morphospecies in non-flowering plants (with four exclusive).

**Figure 4:**
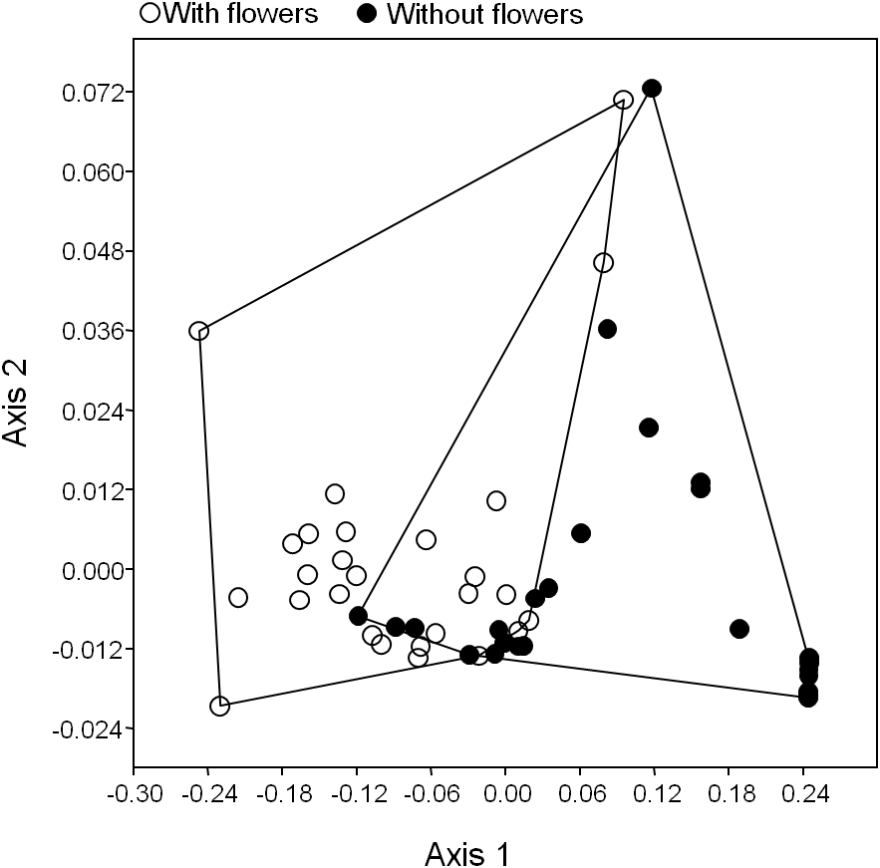
Species composition of phytophagous insects among flowering and non-flowering plants in a Brazilian Caatinga. Morphospecies composition of arboreal insects on trees of *P. pyramidalis* with flowers and without flowers. The dots represent the individual trees and the distance between points represents greater or less dissimilarity between the samples. So the closer the dots, the more similar they are. White dots represent flowering plants and black dots represent trees without flowers.

Regardless of the presence of flowers, higher levels of LCI in leaves positively affected the abundance of phytophagous insects (*F* = 5.44, *p* = 0.02), but not the richness (*F* = 2.6, *p* = 0.11). There was a significant difference in functional groups composition between flowering individuals and those without flowers (ANOSIM, *R* = 0.34, *p* = 0.001; Fig. 4).

## Discussion

The present study demonstrates that flowering trees had higher LCI content and harboured seven times more wood-borers, leaf-chewers and sap-sucking insects than trees without flowers. Our results corroborate the hypothesis of resource concentration originally proposed by Root (1973), where the more herbivorous insects are attracted to areas with a greater availability of high-quality resources. This trend is most evident when we observe that only flowering trees harboured leaf-chewing insects, all belonging to the Chrysomelidae family (Table S1). Flowering plants of *P. pyramidalis* thus demonstrated better nutritional status, indicating that they represent a mosaic of high-quality resources, which determines the distribution of insect diversity during the dry season in our study area.

Habitat complexity and host-plant quality have often been used as ecological variables to explain variation in herbivorous-insect diversity (Espírito-Santo et al., 2007; Leal et al., 2016). As habitat complexity increases, the supply of food resources, space and shelters also increases, leading to a greater diversity of microhabitats compared to plants without reproductive structures (Souza & Módena, 2004). It is clear that inflorescences attract a large number of herbivorous insects and pollinators, providing not only the complexity of the microhabitats but also the supply of nectar, pollen and other food resources (Bairstow et al., 2010; Pearse & Karban, 2013); however, this study demonstrated that flowering plants were also able to attract a higher abundance of insects not directly associated with flower consumption, probably due to the nitrogen content in leaves and branches. For feeders such as sap-sucking and wood-boring insects, higher chlorophyll content may also be associated with a greater palatability of plant tissues.

Many species of phytophagous insects have a long life-history stage with the host plant, indicating that plant characteristics, such as ontogeny and the senescence of tissues, are crucial for them during their entire life-cycle (Hochuli, 2001). Previous studies have indicated that plants may undergo significant changes in leaf nutrient content during leaf senescence (Lee et al., 2003; Milla, Maestro-Martínez & Montserrat-Martí, 2004), affecting foraging behaviour in late season species. Plant phenology can thus be a key factor in maintaining the diversity of arboreal insects, and traits related to resource quality, such as leaf age, play a crucial role in herbivory patterns, with new leaves being more susceptible to herbivorous attack than old ones (Coley, 1980). As deciduous plants, such our study species *P. pyramidalis*, present seasonality associated with the production and fall of leaves (Reich, 1995), the quality (age and senescence) of leaves among flowering and non-flowering plants did not differ. Chlorophyll content would be a factor in our area of study that could explain the higher presence of phytophagous insects in flowering plants. Blooming plants are likely to act as a nitrogen oasis during the dry season.

There is a positive correlation between the nitrogen content in a plant and the leaf chlorophyll content (Schadchina & Dmitrieva, 1995), which probably explains why even non-flowering plants with higher LCI had a greater species richness and abundance of bark and wood-boring insects. Insects prefer plant tissues rich in nitrogen, since it is a limiting factor for development, and production of eggs by females (Eubanks & Styrsky, 2005; Coelho, Veiga & Torres, 2009; Madritch & Lindroth, 2015). These nitrogen-rich tissues can be used by females as preferred oviposition sites, since egg laying occurs on sites (leaves or stems) used by the larval phase (Lara, 1991). In addition to serving as foraging sites, host plants with better food resources are therefore also preferred as oviposition sites (Coll, 1998). In agreement with previous studies, our study also demonstrated that the increase in chlorophyll content was positively associated with insect abundance, especially the woody-borer *Sibinia* sp. The results found here are particularly important when considering the great gap that exists in our knowledge about the fauna and flora of the Caatinga. Due to environmental limitations, the Caatinga has a lower diversity of insect species compared to other environments in Brazil, despite a high rate of endemism and beta diversity (Sousa-Souto et al., 2014; Leal et al., 2016).

Given the pace of anthropogenic impacts in the Caatinga, and the limits on the resources available for conservation (Oliveira & Bernard, in press), quick and cost-effective methods to measure biodiversity are required. Identifying environmental features that reflect the distribution, species richness and abundance of organisms are of clear value to ecologists and conservation managers. Attention should therefore be paid to plant phenology when sampling in future studies, since phenology can indicate not only the quantity and variety of food resources (individuals with blossoms have more appeal than those without blossoms), but also the plant’s nutritional status, as it affects the community structure of the arboreal insects.

## Conclusion

Phytophagous insects choose their feeding resources according to their own requirements. In semiarid environments such as the Caatinga, the high irregularity of rains can cause local exceptional events of flowering in woody plants. Those flowering plants showed higher levels of leaf chlorophyll and consequently harbored a greater abundance of phytophagous insects not directly associated with flowers, such as wood-borers, sap-sucking and leaf-chewing species, indicating that during feeding selection, such insects can detect patches in the landscape with plants of high-nutritional status. Although presenting high endemism, the Caatinga has a low diversity of arboreal insects compared to other tropical ecosystems, and future studies should thus consider host plant phenology during insect sampling, avoiding an underestimation of the local diversity of arboreal insects.

##  Supplemental Information

10.7717/peerj.5059/supp-1 Data S1 Raw data Output of all analyses performed.

10.7717/peerj.5059/supp-2 Table S1 The arboreal insect morphospecies sampled divided in LC, leaf-chewing; SS, sap-sucking; WB, bark and wood-boring insects List of Arboreal insect morphospecies sampled in trees with and without flowers in an area of Caatinga, Brazil. Functional groups: LC, leaf-chewing; SS, sap-sucking; WB, bark and wood-boring insects.

## Additional Information and Declarations

### Field Study Permissions

The following information was supplied relating to field study approvals (i.e., approving body and any reference numbers): Field experiments were approved by SEMARH/SE, Sergipe, Brazil (field permit #2011.04.1008/00123-021/SEMARH/SE).

### Data Availability

The following information was supplied regarding data availability: The raw data are provided in the Supplemental Files.
