# Supplementary material for: Changes in leaf chlorophyll content associated with flowering and its role in the diversity of phytophagous insects in a tree species from a semiarid Caatinga
Source: PeerJ. 2018 Jun 29;6:e5059. doi: 10.7717/peerj.5059 (PMC6055590; doi:10.7717/peerj.5059)
Supplement: Table S1 — List of Arboreal insect morphospecies sampled in trees with and without flowers in an area of Caatinga, Brazil. Functional groups: LC, leaf-chewing; SS, sap-sucking; WB, bark and wood-boring insects. [file peerj-06-5059-s002.docx]

Table S1: List of Arboreal insect morphospecies sampled in trees with and without flowers in an area of Caatinga, Brazil. Functional groups: LC – leaf-chewing, SS – sap-sucking, WB – bark and wood-boring insects.

| Order/Family | Morphospecies | Functional  Group | Flowering plants | Non-flowering plants |
| --- | --- | --- | --- | --- |
| **Coleoptera** |  |  |  |  |
| Bruchidae | Bruchidae sp.1 | WB | 8 | 0 |
|  | Bruchidae sp.2 | WB | 1 | 0 |
|  | Bruchidae sp.3 | WB | 1 | 0 |
|  | Bruchidae sp.4 | WB | 1 | 0 |
|  | Bruchidae sp.5 | WB | 1 | 0 |
| Chrysomelidae | Chrysomelidae sp.1 | LC | 3 | 0 |
|  | Chrysomelidae sp.2 | LC | 4 | 0 |
|  | Chrysomelidae sp.3 | LC | 1 | 0 |
|  | Chrysomelidae sp.4 | LC | 2 | 0 |
|  | Chrysomelidae sp.5 | LC | 1 | 0 |
|  | Chrysomelidae sp.6 | LC | 2 | 0 |
|  | Chrysomelidae sp.7 | LC | 1 | 0 |
| Curculionidae | Curculionidae sp.1 | WB | 4 | 1 |
|  | *Sibinia* *hirritus* | WB | 295 | 47 |
|  | *Sibinia* sp. | WB | 2662 | 415 |
| Anobiidae | *Lasioderma sp.* | WB | 0 | 1 |
|  | *Ptinus* sp. | WB | 0 | 1 |
| Limnichidae | Limnichidae sp.1 | WB | 4 | 0 |
| Scolytidae | Scolytidae sp.1 | WB | 0 | 3 |
|  | Scolytidae sp.2 | WB | 2 | 0 |
| **Hemiptera** |  |  |  |  |
| Cicadelidae | Cicadelidae sp.1 | SS | 9 | 1 |
| Largidae | Largidae sp.1 | SS | 2 | 0 |
| Membracidae | *Acanthuchus* sp. | SS | 0 | 3 |
|  | Membracidae sp.2 | SS | 1 | 0 |
|  | Membracidae sp.3 | SS | 1 | 0 |
| Miridae | Miridae sp.1 | SS | 1 | 0 |
|  | Miridae sp.2 | SS | 1 | 0 |
